# Biochemical, Biophysical and IgE-Epitope Characterization of the Wheat Food Allergen, Tri a 37

**DOI:** 10.1371/journal.pone.0111483

**Published:** 2014-11-04

**Authors:** Sandra Pahr, Regina Selb, Milena Weber, Margarete Focke-Tejkl, Gerhard Hofer, Andela Dordić, Walter Keller, Nikolaos G. Papadopoulos, Stavroula Giavi, Mika Mäkelä, Anna Pelkonen, Verena Niederberger, Susanne Vrtala, Rudolf Valenta

**Affiliations:** 1 Division of Immunopathology, Department of Pathophysiology and Allergy Research, Center of Pathophysiology, Infectiology and Immunology, Medical University of Vienna, Vienna, Austria; 2 Christian Doppler Laboratory for the Development of Allergen Chips, Medical University of Vienna, Vienna, Austria; 3 Department of ENT, Medical University of Vienna, Vienna, Austria; 4 Institute of Molecular Biosciences, Karl-Franzens University Graz, Graz, Austria; 5 Allergy Department, 2nd Pediatric Clinic, University of Athens, Athens, Greece; 6 Skin and Allergy Hospital, Helsinki University Central Hospital, Helsinki, Finland; Cincinnati Children's Hospital Medical Center, University of Cincinnati College of Medicine, United States of America

## Abstract

Wheat is an important staple food and potent allergen source. Recently, we isolated a cDNA coding for wheat alpha-purothionin which is recognized by wheat food allergic patients at risk for severe wheat-induced allergy. The purpose of the present study was the biochemical, biophysical and IgE epitope characterization of recombinant alpha-purothionin. Synthetic genes coding for alpha-purothionin were expressed in a prokaryotic system using *Escherichia coli* and in a eukaryotic expression system based on baculovirus-infected Sf9-insect cells. Recombinant proteins were purified and characterized by SDS-PAGE, mass spectrometry, circular dichroism, chemical cross-linking and size exclusion chromatography. Five overlapping peptid were synthesized for epitope mapping. Alpha-purothionin-specific rabbit antibodies were raised to perform IgE-inhibition experiments and to study the resistance to digestion. The IgE reactivity of the proteins and peptides from ten wheat food allergic patients was studied in non-denaturing RAST-based binding assays. Alpha-purothionin was expressed in the prokaryotic (EcTri a 37) and in the eukaryotic system (BvTri a 37) as a soluble and monomeric protein. However, circular dichroism analysis revealed that EcTri a 37 was unfolded whereas BvTri a 37 was a folded protein. Both proteins showed comparable IgE-reactivity and the epitope mapping revealed the presence of sequential IgE epitopes in the N-terminal basic thionin domain (peptide1:KSCCRSTLGRNCYNLCRARGAQKLCAGVCR) and in the C-terminal acidic extension domain (peptide3:KGFPKLALESNSDEPDTIEYCNLGCRSSVC, peptide4:CNLGCRSSVCDYMVNAAADDEEMKLYVEN). Natural Tri a 37 was digested under gastric conditions but resistant to duodenal digestion. Immunization with EcTri a 37 induced IgG antibodies which recognized similar epitopes as IgE antibodies from allergic patients and inhibited allergic patients' IgE binding. Reactivity to Tri a 37 does not require a folded protein and the presence of sequential IgE epitopes indicates that sensitization to alpha-purothionin occurs via the gut. Both allergens can be used for *in-vitro* diagnosis of wheat food allergy. The induction of blocking IgG antibodies suggests the usefulness for immunotherapy.

## Introduction

Wheat is one of the most important components of our daily nutrition. It contains several essential dietary constituents such as carbohydrates, fats, dietary fibers, minerals, proteins, vitamins and water. [1] However, wheat also belongs to the most important allergy-eliciting foods causing local manifestations as well as severe systemic reactions. [2] In fact, in a population of wheat food allergic children more than 50% had experienced anaphylaxis upon wheat ingestion [3] and wheat has been reported as one of the major food allergen sources involved in food-induced anaphylaxis in a study analysing 1000 patients with food allergy. [4] However, only a few allergen molecules have been identified to be involved in severe allergy to wheat. Among the known wheat food allergens, ω_5_-gliadin (Tri a 19) [5], α/β-gliadins (Tri a 21) [6] and HMW glutenin (Tri a 26) [7] have been described as markers for wheat-dependent exercise-induced anaphylaxis. Furthermore, LTP (lipid transfer protein; Tri a 14) [8] was reported to play an important role in the development of wheat-induced anaphylaxis.

Very recently we identified a novel wheat food allergen, alpha purothionin, which according to the allergen nomenclature system was designated Tri a 37. In 20% of wheat food allergic patients we found IgE reactivity to Tri a 37 which was associated with a four-fold increased risk of experiencing severe wheat-induced anaphylaxis. [9]

The latter finding suggested that Tri a 37 may be a true wheat food allergen (class I food allergen). [9] Class I food allergens usually contain sequential epitopes and sensitization occurs mainly via the gastrointestinal tract. [10], [11] The wheat allergens ω_5_-gliadin, the HMW glutenins [7], [12] as well as allergens from other food allergen sources, such as egg (ovomucoid) [13] and cow's milk (alpha-, beta-casein) are examples for class I food allergens. [14]

Here we report the recombinant expression of Tri a 37 in a prokaryotic system using *Escherichia coli* cells (EcTri a 37) and in an eukaryotic system (baculovirus infected insect cells - BvTri a 37) which yielded an unfolded and a folded form of Tri a 37 and thus allowed us to study the potential relevance of conformational IgE epitopes. Both allergens were characterized regarding biochemical and biophysical properties. A detailed IgE epitope mapping of Tri a 37 was performed with synthetic peptides spanning the complete Tri a 37 sequence. Furthermore, we raised a rabbit antiserum against Tri a 37 to study the natural Tri a 37 under conditions of gastric and duodenal digestion in wheat extract and performed IgE-inhibition experiments to investigate the protective activity of IgG antibodies induced by immunization with Tri a 37.

## Materials and Methods

### Recombinant production of EcTri a 37

The cDNA coding for the mature Tri a 37 with a 3′ sequence coding for a hexahistidine tag was produced as synthetic gene with codons optimized for expression in *E.coli* and subcloned into the pET17b expression vector (ATG-biosynthetics, Merzhausen, Germany). The recombinant allergen was then expressed in *E.coli* BL21 (DE3) cells and purified by nickel affinity chromatography from the inclusion body fraction (Quiagen, Hilden, Germany). [15] The purified recombinant allergen EcTri a 37 was eluted from the column in 8 M urea, 100 mM NaH_2_PO_4_, 10 mM Tris, pH 4.6, then dialyzed step-wise against 10 mM NaH_2_PO_4_ buffer pH 4.0 in which it remained soluble up to 2 mg/ml and could be stored at −20°C. Specific rabbit antibodies against alpha purothionin were raised by immunization of a New Zealand white rabbit with purified EcTri a 37 (200 µg per injection) using once Freund's complete adjuvant and twice Freund's incomplete adjuvant. (Charles River, Kisslegg, Germany). Pre-immune serum was obtained from the rabbit before immunization.

### Recombinant production of BvTri a 37

#### Subcloning of Tri a 37 and production of recombinant bacmid DNA

The cDNA (364 bp) coding for Tri a 37 (accession number AFQ60540.1) containing an additional 3′ sequence coding for a hexahistidine tag was produced as synthetic gene and subcloned into the BamHI/SmaI sites of pUC57 (GenScript, NJ, USA) with codons optimized for insect cell expression. This cDNA was then cloned into the BamHI/SmaI sites of the pTM1 vector harbouring the baculoviral polyhedrin promoter sequence. [16] The plasmid containing the Tri a 37 insert was transformed into XL-1 Blue competent cells (Agilent technologies, Santa Clara, USA) and selected for ampicillin and tetracycline. The correct insertion of the cDNA was confirmed by restriction analysis of plasmid mini-prep DNA (Promega, Madison, Wisconsin, USA) with BamHI/SmaI and by sequence analysis (VBC Biotech, Vienna, Austria). Following the manual instructions (Version A, 15 December 2008) of the Bac-to-Bac TOPO Expression System (Life Technologies, Carlsbad, CA, USA), the recombinant donor plasmid containing the proper DNA sequence was transformed into competent DH10Bac *E.coli* cells, which allow the transposition of the DNA of interest into a baculoviral bacmid. Using blue/white selection and screening for resistance to kanamycin, tetracycline (ROTH, Karlsruhe, Germany) and gentamicin (Life Technologies, Carlsbad, CA, USA) colonies containing the Tri a 37 insert within the bacmid DNA were identified. The presence of the correct recombinant bacmid DNA containing the Tri a 37 gene was confirmed by PCR analysis. A white colony was inoculated in 100 µl water and heated for 5 min at 95°C. PCR analysis was performed using M13 primers (VBC Genomics) and GoTaq DNA polymerase (Promega, Madison, Wisconsin, USA). After midi-prep (Promega) of a positive overnight culture, occurrence of the correct DNA insert was again confirmed by PCR.

#### Production of recombinant baculovirus, determination of viral titer

Recombinant baculovirus was produced using the Bac-to-Bac insect cell expression system (Life Technologies). The recombinant bacmid DNA containing the Tri a 37 DNA was transfected into Sf9 insect cells (Life Technologies) in order to produce recombinant baculovirus. Sf9 insect cells were grown in Sf-900 II SFM media (Life Technologies) supplemented with 10 µg/ml gentamicin (Life Technologies), 2.5% Fetal Bovine Serum (Life Technologies) and maintained in the log phase (3×10^5^–3×10^6^ cells/ml) at 27°C for the entire experiment. For transfection, a total of 2×10^6^ Sf9 insect cells in 2 ml media per well were seeded in a 6-well-plate and incubated for 30 min at 27°C for cell attachment. After replacing the medium containing gentamicin and fetal bovine serum with medium, 100 µl transfection cocktail consisting of 6 µl transfection reagent (FuGENE HD Transfection Reagent, Promega, Madison), 2 µg recombinant bacmid DNA, H_2_O ad 100 ∘l were added to each well. After 7hrs, the medium was removed, replaced with fresh medium containing gentamicin and FBS and incubated for 72hrs at 27°C. Baculoviral stock (P1 viral stock) was collected from the supernatant after centrifugation at 750 xrpm for 5 min at room temperature and stored at 4°C in the dark until use. To determine the viral titre, a plaque assay was performed seeding 2 ml of Sf9 cells (1×10^6^ Sf9 cells/well) in 6-well-plates for 1 hour. After removing medium from the cells, attached insect cells were infected with 1 ml/well of serial viral dilutions (P1: 10^4^, 10^5^, 10^6^; P2 and P3: 10^5^, 10^6^, 10^7^, 10^8^), using 1xDPBS (Life Technologies, Carlsbad, CA, USA), for 1 hour at room temperature. The supernatant was removed and infected insect cells were covered with 2 ml of Sf-900 medium 1.3X (Life Technologies) containing 1% low-melting agarose and 10 µg/ml of gentamicin. Plates were kept, without moving, for 1 hour at room temperature allowing the agarose mixture to solidify. After wrapping the plate in moisturized paper, the plate was covered with aluminium foil and incubated for 5–7 days at 27°C. Plaques were stained with 2 ml of neutral red solution (Sigma Aldrich, Munich, Germany), diluted 1∶10 in 1xPBS for 2 hours at room temperature. After removal of the staining solution, plaques became visible and were counted. For further amplification of P1 stock to generate a high-titer P2 stock, the following formula was used: inoculum required (ml)  =  [MOI (pfu/cell) x number of cells]/titer of viral stock (pfu/ml). Ten ml of cells (2×10^6^ cells/ml → 2×10^7^ cells) were infected with 2 ml of P1 stock (1×10^6^ pfu/ml) to obtain a MOI (multiplicity of infection: number of virus particles per cell) of 0.1. The viral titer of P2 stock was again determined by plaque assay and stored as described above for P1 viral stock.

In optimization experiments, several conditions (MOI 1, 2, 3, 4; durations of expression 24hrs, 48hrs, 72hrs, 96hrs) were tested to optimize the protocol. Samples were taken, centrifuged at 10,000 rpm for 2 min, sample buffer was added to the supernatants and stored at 4°C. All collected samples were subjected to 14% SDS-PAGE, blotted onto nitrocellulose and tested with Tri a 37-specific rabbit antibodies to detect the level of BvTri a 37 expression.

#### Expression of BvTri a 37

For expression of BvTri a 37, 200 ml of 2×10^6^ cells/ml Sf9 cells present in fresh Sf-900 II SFM medium (+Gm, -FBS) were infected with the viral P2 stock to reach MOI = 1 for 96hrs while shaking at 80 rpm/min at 27°C in the dark. After centrifugation at 750 xrpm for 15 min, PMSF (10 µg/ml) were added to secreted protein-containing supernatants. For protein purification under native conditions, using nickel affinity chromatography (Qiagen), supernatants were dialyzed against lysis buffer (50 mM NaH_2_PO_4_, 300 mM NaCl, 1 mM Imidazole, pH 8.0) o/N, and incubated with pre-equilibrated nickel agarose in a batch procedure o/N. The agarose batch was filled into column, washed with wash buffer (50 mM NaH_2_PO_4_, 300 mM NaCl, 20 mM Imidazole, pH 8.0) and eluted with elution buffer (50 mM NaH_2_PO_4_, 300 mM NaCl, 250 mM Imidazole, PH 8.0). BvTri a 37-containing fractions were pooled and dialyzed against 10 mM NaH_2_PO_4_ pH 8.0 and stored at −20°C.

### Characterization of recombinant proteins

The protein concentrations of both recombinant allergens were determined in parallel by BCA assay (Pierce, Rockford, IL) and purity was assessed by Coomassie Blue-stained 14% SDS–PAGE. The protein was analysed under reducing and non-reducing conditions. [17] Nitrocellulose-blotted, recombinant EcTri a 37 and BvTri a 37 (2 µg each) were tested with serum (dilution 1∶10) from a wheat food allergic patient (Patient 1, Table 1) and buffer as a control. Bound IgE antibodies were detected with ^125^I-labeled anti-human IgE antibodies (Demeditec Diagnostics, Kiel, Germany) and visualized by autoradiography. Aqueous wheat seed extract (20 µg) was blotted and probed with Tri a 37-specific rabbit antibodies and for control purposes with the corresponding pre-immune serum (dilution 1∶10.000). Bound IgG antibodies were detected with ^125^I-labeled goat anti-rabbit IgG antibodies (Perkin Elmer, Boston, USA) and visualized by autoradiography. The identity of the recombinant proteins was confirmed by mass spectrometry (Bruker, Billerica, MA). [18] Mass spectrometry, size exclusion chromatography and circular dichroism measurement (CD) were performed as previously described. [18] For the chemical crosslinking of oligomers in solution 2 ng of EcTri a 37 or BvTri a 37 were incubated in a total volume of 15 µl with 0.01–0.2% v/v glutaraldehyde for 15 minutes at room temperature. To generate samples in a reduced condition 10 mM DTT was added 10 minutes prior to the glutaraldehyde. The reaction was stopped by adding 2 µl of a 1 M glycine solution. The resulting products were assessed by 18% SDS-PAGE and by size exclusion chromatography. [18]

**Table 1:** Demographic, clinical and serological characterization of wheat food-allergic patients.

Patient	Age	Sex	Wheat-inducedSymptoms	Wheat-inducedanaphylaxis	Total IgE(kU/L)	Wheat-specific IgE(kUA/L)	Wheat SPTMean wheal diameter (mm)	Wheatchallenge	omega-5-gliadin(kUA/L)	Tri a 36	Reactionafter
1	41	F	AS, GIS	yes	252	180	pos	n.d.	9.66	+	wheat-containing products
2	5	M	SS	no	7559	45.2	6	open (pos)	2.37	+	open challenge
3	14	M	AS, SS	yes	636	>200	8	open (pos)	n.d.	+	bread
4	3	M	GIS, SS	yes	165	11.7	8	n.d.	0.22	+	pasta soup, baby cereals
5	10	M	AS, GIS	yes	802	456	7	n.d.	20.3	+	cereals
6	11	M	GIS	no	1642	519	12	open (pos)	3.33	+	open challenge
7	22	M	GIS	no	n.d.	47	6	open (pos)	0.46	+	open challenge
8	4	F	AS, SS, GIS	yes	483	66.3	4.5	open (pos)	4.56	+	open challenge
9	6	F	AS, SS	yes	74	7.57	6	open (pos)	0.04	+	open challenge
10	3	F	SS	no	59.4	3.1	3	n.d.	0.01	-	wheat soup

Abbreviations: kU/L: kilounit per liter, kUA/L: kilounit antigen per liter, AS: airway symptoms, GIS: gastrointestinal symptoms, SS: skin symptoms, n.d.: not done, SPT: skin prick test.

Tri a 37 was analyzed using the Expasy ProtScale accessibility program for the presence of surface-exposed areas. [19]

### Sera from wheat allergic patients used for testing the IgE reactivity of the allergens

Sera were obtained from European wheat food allergic patients (Austria n = 1; Finland: n = 3; Greece: n = 6; total: n = 10) aged 3–41 years (mean age 12 years). Patients were diagnosed on the basis of a case history demonstrating that allergic symptoms (airway symptoms, gastrointestinal symptoms, skin symptoms, systemic anaphylaxis) were unambiguously related to the ingestion of wheat or wheat-containing products. A grading of symptoms and definition of anaphylaxis was performed according to the international position paper and recommendations for the definition of anaphylaxis (Table 1). [20] In six of the patients, open food challenge was performed and positive challenge results were obtained. Skin prick tests with wheat seed extract were performed and positive in each of the wheat allergic patients. For each patient IgE-mediated sensitization to wheat was confirmed by measurements of allergen-specific IgE (CAP-FEIA, Thermofisher, Uppsala, Sweden). Patients were further characterized for their reactivity to the two major wheat food allergens omega-5-gliadin (Tri a 19) and LMW glutenin (Tri a 36). [21]

### Ethical Considerations

Serum samples were obtained in the course of routine allergy diagnosis with permission of the Helsinki University Hospital Review Board/Section for Pediatrics (Dnro 374/E7/2004), Regional Committee of Ethics of the Kopodistrian University of Athens (289/2007) and the Ethics Committee of the Medical University of Vienna (565/2007). Written informed consent was obtained from adult patients and in case of children, from their parents. A retrospective analysis of allergen-specific IgE antibodies was performed with anonymized serum samples with permission of the Ethics Committee of the Medical University of Vienna.

### Determination of IgE-reactivity by dot-blotting

IgE-reactivity of EcTri a 37, BvTri a 37 and five peptides (P1–P5) was tested by by non-denaturing, RAST-based IgE dot blotting. [15] For dot blotting, aliquots of aqueous wheat seed extract (2 µg/dot), HSA (0.5 µg/dot), recombinant EcTri a 37, BvTri a 37 and peptides (0.5 µg/dot) were dotted onto nitrocellulose strips (Schleicher & Schuell, Dassel, Germany). Strips were incubated with 1∶10 diluted sera of allergic patients sera (1–10) or with 1∶10,000 diluted EcTri a 37-specific rabbit antibodies. As a control, serum of a non-atopic individual (N), rabbit pre-immune serum or buffer (B) alone were included. Bound IgE antibodies were detected with ^125^I-labeled anti-human IgE antibodies (Demeditec Diagnostics, Kiel, Germany), bound rabbit antibodies with ^125^I-labeled anti-rabbit IgG antibodies (Perkin Elmer, Waltham, USA) and visualized by autoradiography. [22]

### Synthesis, characterization and antibody reactivity of Tri a 37 peptides

Five 10aa- overlapping peptides spanning the Tri a 37 sequence (Table 2; accession number AFQ60540.1) were synthesized (Microwave peptide synthesizer liberty CEM GmbH, Germany) using a Fmoc (9-fluorenylmethoxycarbonyl)-strategy with HBTU [2-(1H-Benzotriazol-1-yl)1,1,3,3 tetramethyluronium hexafluorophosphat] activation as described by Focke *et al*. [23] Table 2 shows the amino acid sequences of Tri a 37-derived peptides. Four of the five peptides had a length of 30 amino acids whereas peptide 4 was synthesized without C-terminal cysteine and hence only 29 aa long. After purification by HPLC to a purity >90%, the identities of the peptides were confirmed by mass spectrometry.

**Table 2:** Characterization of Tri a 37-derived peptides and of the two Tri a 37 domains.

Peptides	Sequence	Molecular weight	Isoelectric point
**P1**	KSCCRSTLGRNCYNLCRARGAQKLCAGVCR	3291.91	9.69
**P2**	AQKLCAGVCRCKISSGLSCPKGFPKLALES	3095.75	9.21
**P3**	KGFPKLALESNSDEPDTIEYCNLGCRSSVC	3276.66	4.51
**P4**	CNLGCRSSVCDYMVNAAADDEEMKLYVEN	3243.62	4.02
**P5**	EEMKLYVENCADACVSFCNGDAGLPSLDAY	3228.58	3.71

		Thionin domain (45 aa)	C-terminal acidic extension domain (65 aa)
	**basic aa**	22.20%	3.10%
	**acidic aa**	0%	22%
	**pI**	9.75	3.35
	**hydrophobic aa**	18%	22%
	**hydrophilic aa**	60%	48%

### Inhibition of allergic patients' IgE-binding to EcTri a 37 by rabbit IgG antibodies as determined by ELISA inhibition assay

ELISA plates (Nunc-Immuno Plate, Maxisorp, Thermofisher Scientific, Denmark) were coated with EcTri a 37 diluted in PBS (c = 2 µg/ml) overnight at 4°C. After washing plates twice with PBST and blocking with blocking buffer (PBST, 1% [w/v] BSA) for 2 hours at room temperature, plates were incubated overnight at 4°C with rabbit anti-EcTri a 37 immune-serum or the corresponding pre-immune serum which had been diluted 1∶50 in PBST, 0.5% (w/v) BSA. After washing, plates were incubated with 1∶10 diluted sera from wheat food allergic patients (Table 1; Patients 1, 3, 4) overnight at 4°C and bound human IgE antibodies were detected with horseradish peroxisase (HRP)-coupled goat anti-human IgE antibodies (KPL, Gaithersburg, MD) diluted 1∶2,500 in PBST, 0.5% (w/v) BSA. The color reaction was induced by addition of 1.7 mM 2,2′-azino-di-[3-ethyl-benzthiezolin-sulfonet] (Sigma-Aldrich) in 60 mM citric acid, 77 mM Na_2_HPO_4_·2H_2_O and 3 mM H_2_O_2_.OD measurements were performed after 30 minutes in an ELISA reader (Spectra Max PLUS 384, Molecular Devices, Germany) at 405 nm-490 nm. The percentage of inhibition of IgE binding was calculated as follows: 100-(ODimmune/ODpre)x100, where ODimmune and ODpre represent the extinction coefficients after pre incubation with the immune serum or with the pre-immune serum, respectively.

### *In-vitro* digestion assays

Gastric and duodenal *in-vitro* digestion assay with wheat seed extract was performed as described. [24], [25] Aliquots containing 10 µg undigested or digested extract were loaded on SDS-PAGE and blotted onto nitrocellulose membranes. Membranes were incubated either with rabbit Abs raised against EcTri a 37 or the corresponding pre-immune serum. Bound rabbit Abs were detected with ^125^I-labeled anti-rabbit IgG Abs (Perkin Elmer, Waltham, USA) and visualized by autoradiography.

## Results

### Expression of recombinant Tri a 37 proteins as unfolded and folded protein

**Figure 1:**
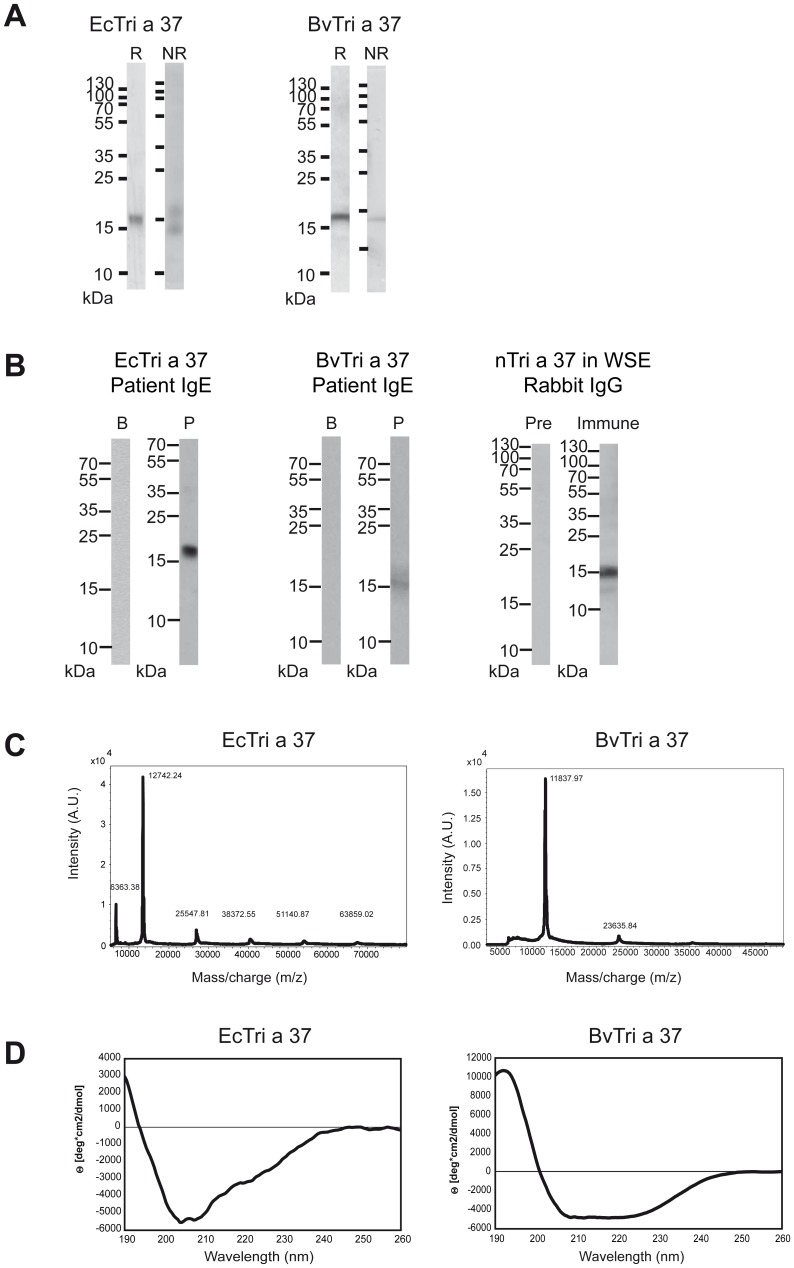
Characterization of purified Tri a 37 expressed in *E.coli* cells (EcTri a 37) and in baculovirus-infected insect cells (BvTri a 37) as well as detection of natural Tri a 37 in wheat seed extract. (**A**) Coomassie brilliant blue-stained SDS-PAGE under reducing (lane R) and under non-reducing (lane NR) conditions. A protein molecular weight marker (kDa) is shown on the left side. (**B**) Detection of IgE-reactivitiy to Western-blotted EcTri a 37 and BvTri a 37, using serum from a wheat food allergic patient (P) and buffer (B) as a control. Western-blotted wheat seed extract was tested with Tri a 37-specific rabbit antibodies (Immune) or the corresponding pre-immune serum (Pre). Bound human IgE and rabbit IgG antibodies were detected with ^125^Iodine-labeled antibodies and visualized by autoradiography. Molecular weights are indicated in kilo Dalton (kDa). (**C**) Mass spectrometry of recombinant EcTri a 37 and BvTri a 37. The mass/charge ratios are shown on the *x*-axes, and the intensities are displayed on the *y*-axes as percentages of the most intensive signals obtained in the investigated mass range. (**D**) Circular dichroism analysis of recombinant EcTri a 37 and BvTri a 37. The spectra are expressed as mean residue ellipticities (θ) (*y*-axes) at given wavelengths (*x*-axes).

rTri a 37 expressed in a prokaryotic system using *E.coli* cells was designated EcTri a 37, however rTri a 37 expressed in baculovirus-infected insect cells was termed BvTri a 37. Both recombinant proteins were purified by Nickel affinity chromatography and showed a similar migration behaviour in Coomassie Blue-stained SDS-PAGE under reducing conditions where they appeared as bands of approximately 18kDa which differs from their calculated molecular weights (EcTri a 37 including N-terminal methionine and C-terminal hexahistine-tag: 12.757 Da; BvTri a 37 including a D and P residue at the N-terminus and a C-terminal hexahistidine-tag: 12.838 Da) and may be explained by the high content of positively charged amino acids (Figure 1A). Under non-reducing conditions, BvTri a 37 appeared as single band of approximately 14 kDa whereas EcTri a 37 showed an additional second band of approximately 18 kDa but no high molecular weight aggregates were visible in both protein preparations (Figure 1A). Using serum IgE from a wheat food allergic patient, monomeric EcTri a 37 and BvTri a 37 were detected at approximately 18 kDa by immunoblotting. Tri a 37-specific rabbit antibodies reacted with natural, monomeric Tri a 37 at 15 kDa in a nitrocellulose-blotted aqueous wheat seed extract (Figure 1B).

MALDI-ToF analysis of EcTri a 37 resulted in a mass peak of 12.742 Da corresponding to the predicted molecular weight of the monomeric protein including methionine and a hexahistidine-tag. (Figure 1C, left). Three BvTri a 37 preparations were analyzed by MALDI-ToF. For one preparation the molecular weight determined by mass spectrometry (i.e., 11.837 Da) was smaller than the calculated mass (Figure 1B, right) whereas for the two other preparations the measured masses were 12.829 Da and 12.913 Da, respectively, which fitted the calculated mass (i.e., 12.838 Da). The smaller mass of the first preparation was most likely was due to N-terminal proteolytic degradation because the His-tag was intact as shown by immunoblotting with an anti-His-tag antibody (data not shown). Chemical cross-linking experiments and size exclusion experiments showed that both, EcTri a 37 and BvTri a 37, occurred as monomeric proteins (data not shown).

The far-UV CD spectrum of EcTri a 37 indicated that the recombinant protein was unfolded which is characterized by minimum at approximately 205 nm. By contrast, BvTri a 37 appeared as folded protein showing a CD spectrum typical for a predominant α-fold with two minima at 208 and 222 nm and a maximum at approximately 190 nm (Figure 1D).

### Unfolded EcTri a 37 and folded BvTri a 37 show comparable IgE-reactivity

**Figure 2:**
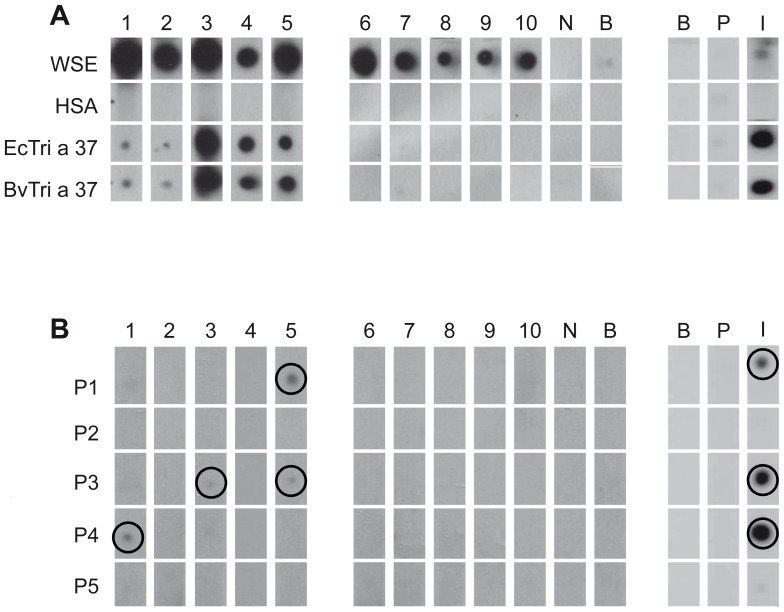
Antibody-recognition of EcTri a 37, BvTri a 37 and Tri a 37 peptides. (**A**) Nitrocellulose-dotted wheat seed extract (WSE), human serum albumin (HSA), EcTri a 37 and BvTri a 37 were tested with sera from ten wheat food allergic patients (1–10), with serum from a non-allergic individual (N), buffer alone (B), with Tri a 37-specific rabbit antibodies (I), the corresponding rabbit pre-immune serum (P) and buffer control (B). (**B**) Testing of Tri a 37 peptides (P1–P5) as in (A).

The IgE-reactivity of both recombinant allergens EcTri a 37 and BvTri a 37 was tested in non-denaturing, RAST-based IgE-dot blot experiments with sera from 10 patients with confirmed wheat-induced food allergy (Figure 2A; Table 1). Both allergens showed very similar and specific IgE-binding capacity when tested with the sera from the five wheat allergic patients who were sensitized to Tri a 37 (patients #1–5) but not with sera from wheat allergic patients who were not sensitized to Tri a 37 (patients #6–10). Also Tri a 37-specific rabbit antibodies reacted with both recombinant allergens in a comparable manner (Figure 2A). Allergic patients' sera, but not serum from a non-allergic subject or buffer displayed IgE reactivity to wheat extract. Likewise, wheat extract was recognized by the rabbit anti-Tri a 37 antibodies but not by the pre-immune serum or buffer. The negative control protein HSA showed no reactivity (Figure 2A).

### Tri a 37 contains sequential IgE epitopes in the N- as well as C- terminal domain

**Figure 3:**
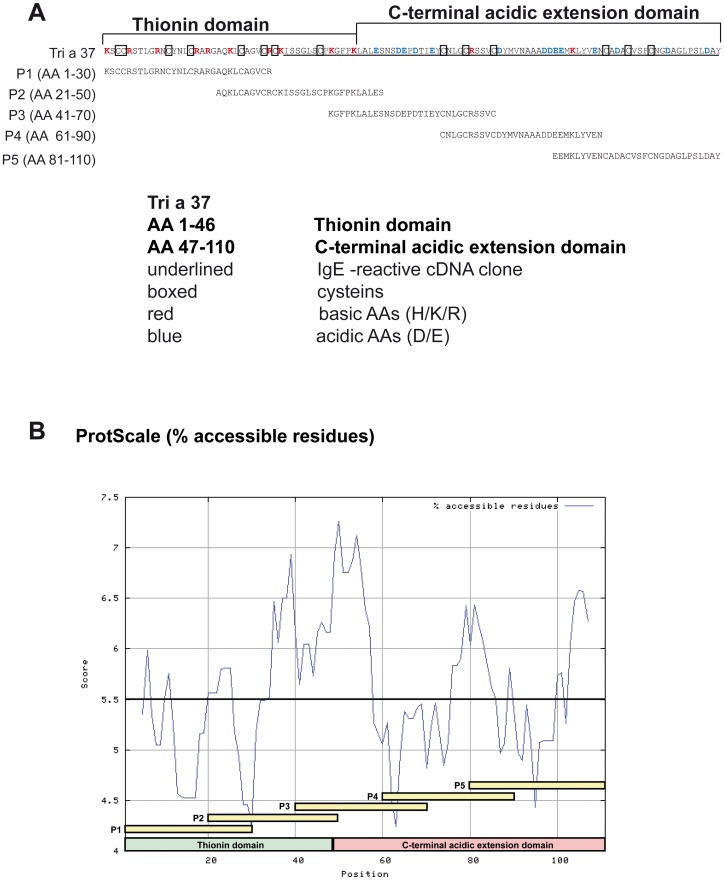
Localization of synthetic peptides in the Tri a 37 sequence and surface accessibility prediction. (**A**) Amino acid sequence of Tri a 37 showing the N- and C-terminal domain and the originally isolated IgE-reactive fragment (underlined). Cysteins are boxed, basic and acidic amino acids are coloured in red and blue, respectively. Synthetic Tri a 37 peptides P1–P5 are indicated. (**B**) Surface accessibility plot of the Tri a 37 amino acid sequence. The numbers of the amino acids, the thionin domain (green), the C-terminal acidic extension domain (pink), the synthetic peptides (yellow) are indicated on the x-axis. The calculated accessibility of the residues is indicated on the y-axis. Scores above 5.5 (bold horizontal line) represent regions of high surface accessibility.

In order to study the IgE epitopes of Tri a 37 in more detail, we tested the sera from the wheat allergic patients also for IgE reactivity with synthetic Tri a 37 peptides (Figure 2B). Five overlapping peptides spanning the Tri a 37 sequence were synthesized and purified (Figure 3, Table 2). With the exception of peptide 4 which was 29 amino acids long, the synthesized peptides were 30 amino acids long with overlaps of ten amino acids (Figure 3A). The molecular weights of the peptides range from 3095 to 3291 Dalton. Three of the synthesized peptides were from the original IgE-reactive recombinant fragment whereas peptides 1 and 2 also included portions of the N-terminal thionin domain which is rich in basic amino acids and has a pI of 9.75 whereas the C-terminal acidic extension domain is acidic (pI 3.53) (Figure 3A; Table 2). Hence, the isoelectric points of the individual peptides ranged from 3.71 to 9.69 depending on the domain from which they originated (Table 2). Peptides 1, 3 and 4 from both domains were recognized by IgE antibodies from Tri a 37-sensitized patients and also by Tri a 37-specific rabbit antibodies. Peptides 2 and 5 did not react with IgE from the tested patients and also not with rabbit IgG antibodies raised by immunization with rTri a 37 (Figure 2B). According to the prediction of surface-exposed portions of Tri a 37, the IgE-reactive peptides were derived from surface-exposed regions (e.g., peptides 3 and 4) but included also less surface-exposed areas (i.e., peptide 1) (Figure 3B).

### IgG antibodies induced by immunization with EcTri a 37 recognize similar epitopes as allergic patients IgE and inhibit allergic patients' IgE binding to Tri a 37

**Figure 4:**
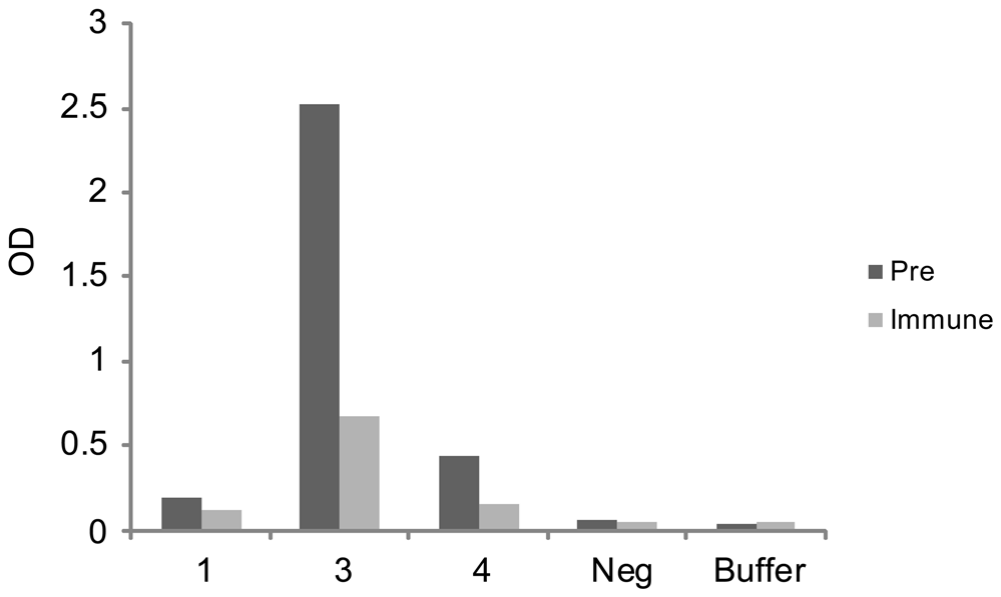
Inhibition of allergic patients' IgE binding to Tri a 37 by rabbit anti-Tri a 37 antibodies. Tri a 37 was tested for IgE reactivity with sera from three Tri a 37-allergic patients (1, 3, 4), serum from a non-allergic individual or buffer after pre-incubation with rabbit anti-Tri a 37 antibodies (Immune) or the rabbits pre-immune serum (Pre). Shown are the optical density (O.D.) levels corresponding to bound IgE.

EcTri a 37- specific rabbit IgG antibodies were then used to investigate their ability to inhibit wheat food allergic patients' IgE binding (Table 1; Patient 1, 3, 4) to the allergen in ELISA competition experiments. In patients 3 and 4 who showed the strongest IgE binding to Tri a 37, rabbit anti-Tri a 37 antibodies caused a 64% and 73% inhibition of IgE binding respectively whereas the inhibition in patient 1 who showed low levels of Tri a 37-specific IgE was 39% (Figure 4).

### Natural Tri a 37 in wheat extracts is digested under gastric digestion conditions

**Figure 5:**
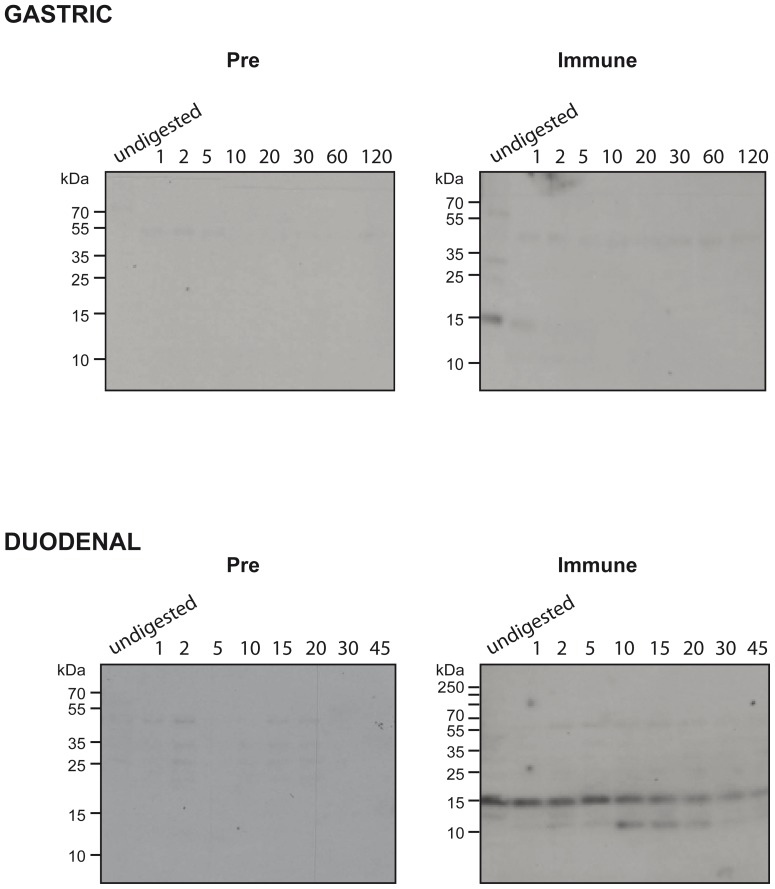
Effects of gastric and duodenal digestion on natural Tri a 37. Undigested wheat seed extract (undigested) or after different times of gastric (top panel) or duodenal digestion (lower panel) was subjected to SDS-PAGE and blotted to nitrocellulose. Nitrocelluloses were then tested with rabbit anti-Tri a 37 antibodies (Immune) and the corresponding pre-immune serum (Pre). Bound IgG antibodies were detected with ^125^I-labeled anti-rabbit IgG antibodies and visualized by autoradiography.

Figure 5 shows nitrocellulose-blotted wheat extracts which were subjected to gastric and duodenal digestion and then probed with rabbit anti-Tri a 37 antibodies. Undigested Tri a 37 was detected at 15 kDa whereas one minute of gastric digestion led to the loss of the signal. Under duodenal digestion conditions, Tri a 37 could be detected even after 45 minutes at 15 kDa and an additional smaller IgG binding band at 10 kDa became visible after 10 minutes of digestion (Figure 5).

## Discussion

Here we showed that IgE recognition of Tri a 37, a wheat food allergen which is associated with severe wheat-induced anaphylaxis does not require conformational epitopes. Using two different expression systems, an unfolded variant of rTri a 37 (i.e., EcTri a 37) was produced by prokaryotic expression and a folded form was obtained by expression in baculovirus-infected insect cells (i.e., BvTri a 37). The differences in fold between the two recombinant proteins were not due to differences in glycosylation because Tri a 37 does not contain any glycosylation sites. However, Tri a 37 contains 14 cystein residues of which 8 are known to form 4 disulphide bridges in a correctly folded N-terminal thionin domain for which the three-dimensional structure was obtained by x-ray crystallography and nuclear magnetic resonance analysis. [26], [27] Interestingly, both, the folded as well as the unfolded Tri a 37 occurred as monomeric proteins in SDS-PAGE under non-reducing conditions and when analyzed by size exclusion chromatography indicating that in both proteins the 14 cysteins were mainly engaged in the formation of intra-molecular but did not form inter-molecular disulphide bonds. The presence and lack of structural fold in the two proteins therefore may be attributed to correct versus partly incorrect formation of intra-molecular disulphide bonds. Comparable IgE binding capacity of EcTri a 37 and BvTri a 37 in non-denaturing, RAST-based IgE binding assay revealed that wheat food allergic patients' IgE recognition does not require structural fold of Tri a 37. A more detailed analysis of IgE epitopes was performed with 5 synthetic peptides spanning the complete Tri a 37 sequence which had an overlap of 9–10 amino acids. Using these synthetic peptides, sequential IgE binding sites were found at the N-terminal thionin domain and the C-terminal extension domain. Not all Tri a 37-reactive sera reacted also with peptides which may be due to the fact that certain IgE-reactive areas were not covered by the peptides due to limited overlap or length. Nevertheless, our data demonstrate that IgE recognition of Tri a 37 thus does not require fold and is directed against sequential epitopes which is typical for class I food allergens which sensitize mainly via the gut such as other wheat food-, milk, egg and peanut allergens. When we digested natural wheat extract containing Tri a 37, we found that the allergen was digested under conditions of gastric digestion whereas it was stable under conditions of duodenal digestion. This finding fits to the observation that Tri a 37 contains sequential epitopes which are represented by peptides. IgE recognition of sequential epitopes might thus be explained by sensitization to proteolytic fragments of Tri a 37. In this context it is tempting to speculate that sensitization to Tri a 37 and symptoms caused by Tri a 37 may be modulated by conditions affecting gastric digestion such as pathologies of the gastrointestinal tract and low acidic conditions which may occur in in children or elderly persons or as consequence of antacid medication. [28], [29]

Currently several approaches of allergen-specific immunotherapy are pursued for food allergies, among them oral, sublingual and epicutaneous immunotherapy with natural food allergens [30] and also injection immunotherapy approaches using recombinant food allergens. [31] In this context, we investigated the ability of rabbit IgG antibodies which were induced by immunization with recombinant Tri a 37, to inhibit allergic patients IgE binding to Tri a 37. In fact, we found that Tri a 37-specific IgG inhibited allergic patients IgE binding to Tri a 37 indicating that it may be possible to perform injection immunotherapy with recombinant Tri a 37 with the goal to induce blocking IgG antibodies which are known to play a major role in immunotherapy.

In conclusion we showed that IgE epitopes of Tri a 37 are of the non-conformational type, including sequential epitopes and that rTri a 37 expressed in *E.coli* as well as insect cells can be used for the serological detection of Tri a 37-specific IgE in diagnostic tests. Furthermore, it may be possible to use rTri a 37 for immunotherapy in sensitized individuals.
